# Comparisons of (cost-)effectiveness of manual, hybrid, and digital shape capture and shape design techniques for transtibial and transfemoral prosthetic sockets: A scoping review

**DOI:** 10.1097/PXR.0000000000000531

**Published:** 2026-03-11

**Authors:** Pien van Gastel, Iris Pieta Jacoba Sterkenburg, Gerwin Smit, Merel van der Stelt, Ruud Leijendekkers

**Affiliations:** 1 3D Lab Radboudumc, Radboud University Medical Center, Nijmegen, The Netherlands; 2 Department of BioMechanical Engineering, Delft University of Technology, Delft, The Netherlands; 3 Department of Rehabilitation, Radboud University Medical Center, Nijmegen, The Netherlands; 4 Radboud Institute for Health Sciences, IQ Healthcare, Radboud University Medical Center, Nijmegen, The Netherlands

**Keywords:** prosthetic socket, transtibial, transfemoral, computer-aided design, (cost-)effectiveness

## Abstract

This scoping review provides an overview of studies comparing the (cost-)effectiveness of shape capture and socket design techniques for transtibial and transfemoral prostheses. The review compares manual, hybrid, and digital methods, identifies the measurement tools used, and assesses their methodological quality. Effectiveness refers to clinical and functional outcomes such as socket fit, comfort, and user function, whereas cost-effectiveness reflects the balance between resource use and these outcomes. Following Preferred Reporting Items for Systematic Reviews and Meta-analysis guidelines, 5 databases (PubMed, Embase, Web of Science, CINAHL, and Cochrane) were systematically searched. Studies involving humans with transtibial or transfemoral prostheses that compared at least 2 of the 3 methods and reported (cost-)effectiveness outcomes were included. Of 556 articles screened, 20 met the inclusion criteria (497 participants). Sixteen studies evaluated transtibial prostheses and 4 transfemoral prostheses. Manual and hybrid methods were compared in 14 studies, and digital and manual methods in 6, whereas none compared hybrid and digital methods. Eighteen studies were rated as low quality, 2 as moderate, and none as high. Effectiveness constructs mainly covered the International Classification of Functioning, Disability and Health domains “Body functions & Body structures” and “Activities and participation,” but many were not clearly defined within this framework. Reported outcomes most often addressed production time, number of socket attempts, and socket fit or comfort. Overall, evidence remains limited and inconsistent, with a clear lack of direct comparisons between digital and hybrid techniques. Tentatively, hybrid and digital approaches may improve efficiency and comfort compared with manual methods, but robust, standardized research is needed to confirm these effects.

## Introduction

In 2005, an estimated 1.6 million people in the United States were living with limb loss, including approximately 0.6 million with major lower-limb loss.^1^ In the Netherlands, 22% of lower limb amputations are performed at the transtibial level, and 14% at the transfemoral level.^2^ Transition to a well-fitting prosthesis can enable a person to regain independence and engage in daily activities again.^3^ A transtibial prosthesis (i.e., below-knee prosthesis) typically consists of a socket, suspension system, and foot. A transfemoral prosthesis (i.e., above-knee prosthesis) includes these components, together with a knee joint. In both prostheses, the socket serves as an integral interface connecting the human body to the prosthesis.

Achieving an optimal socket fit remains one of the main challenges in prosthetic care.^3^ Poor fit can cause excessive limb-socket interface stresses and skin problems,^3,4^ aggravated by limb volume changes and heat accumulation within the socket.^5,6^ These issues reduce function and quality of life and often require frequent socket adjustments, increasing clinical workload and health care costs.^3,7,8^

The process of developing a prosthetic socket involves 3 key phases. First, the shape of the residual limb is captured, referred to here as shape capture. Second, the socket is designed using modifications to obtain a socket with adequate load transfer, referred to here as the socket shape design. Designing the socket consists of different aspects: (1) the socket type (e.g., specific weight-bearing or total surface-bearing socket for transtibial prostheses), (2) the socket shape design (i.e., defining its spatial characteristics and inner surface dimensions in relation to the residual limb shape), and (3) the proximal and distal parts of the socket (i.e., the socket brim and adapter, respectively). Last, the socket is manufactured. This review will focus on the shape capture and socket shape design.

Conventionally, all prosthetic socket developing steps relied on manual methods. During the shape-capturing phase, a plaster cast is applied around the residual limb to create a custom negative mold. This process involves not only capturing limb geometry but also assessing tissue composition, muscle potential, skin condition, sensitivity, and adjacent joint range of motion, which inform decisions about volume reductions, trimline geometry, and load distribution.^9,10^ In the subsequent socket shape design phase, this negative mold is filled with plaster to form a positive mold, which the prosthetist can manually modify. A personalized socket is then manufactured around this mold using methods such as thermo- or vacuum forming.^11^ However, this manual approach heavily depends on the prosthetist’s expertise, requiring specialized knowledge and significant time, while offering limited reproducibility.^12,13^ To improve consistency in limb shaping and load application, various manual casting aids, such as adjustable brims, vacuum jigs, and specialized molding devices, have been developed.^14^

Despite these advancements, manual methods remain time-consuming and operator-dependent. To address these limitations, digital and hybrid methods have been developed. A digital approach to capturing the shape of the residual limb involves using techniques such as 3D scanning, followed by computer-aided design software for patient-specific modifications to the socket shape. This approach eliminates the need for physical molds, although manual modifications in computer-aided design still rely on the prosthetist’s expertise.^15^ Hybrid methods combine both manual and digital techniques for shape capture and socket shape design.

Socket shape design can be categorized into 3 levels of standardization: not standardized, semi-standardized, and fully standardized. Not standardized techniques rely solely on manual modifications by the prosthetist, offering high individualization but limited reproducibility. Semi-standardized methods combine predefined templates or modifications with prosthetist expertise, balancing efficiency and customization. Fully standardized approaches automate the design process, reducing dependence on prosthetist skill and ensuring more consistent outcomes,^16^ although they may limit adaptability for patients with unique anatomical or functional needs. Understanding these distinctions is essential for interpreting subsequent studies on manual, hybrid, and digital socket design approaches.

Previous reviews, such as that by Yang et al,^17^ primarily examined shape capture techniques for transtibial sockets, focusing on manual and digital approaches while excluding studies that integrated socket design or hybrid methods. In contrast, the present review extends this scope to include both transtibial and transfemoral prostheses, incorporates socket shape design techniques, and explicitly compares their (cost-)effectiveness.

Hybrid and digital methods are often expected to offer advantages in (cost-)effectiveness. However, many studies lack comparative analyses with manual methods or rely on finite element analysis, which may not directly translate to clinical applicability.^18^ Furthermore, there is a deficiency in thorough economic evaluations that compare these various methods.^19^ In line with standard health economic definitions, “effectiveness” refers to the extent to which a technique achieves intended clinical and functional outcomes (e.g., socket fit, comfort, user function, and participation), whereas “cost-effectiveness” refers to the relationship between the resources used and the benefits achieved, typically expressed as the additional cost per additional unit of effect.^20^ In this review, because of the limited availability of formal cost-effectiveness studies, we include studies reporting resource use (e.g., production time, number of socket attempts) as a pragmatic proxy for cost-effectiveness, acknowledging that this provides only a partial assessment. This evaluation is critical in assessing the value of novel shape capture and socket shape design methods, along with their standardization level. Moreover, effectiveness extends beyond socket fit, encompassing broader aspects of physical function and well-being, which are inconsistently defined and measured across studies.

Recognizing this gap, there is a need for a comprehensive overview of studies that compare these methods, focusing on their (cost-)effectiveness. Therefore, the aim of this scoping review is 2-fold: (1) to provide an overview of studies comparing the (cost-)effectiveness of manual, hybrid, and digital methods for shape capture and/or shape design techniques for transfemoral and transtibial prosthetic sockets and (2) to identify the measurement constructs and instruments used to assess (cost-)effectiveness in these contexts. By providing an overview of these studies, this review could guide future research directions and inform clinical decision making, thereby contributing to the application of novel methods in the field of prosthetics.

## Methods

### Study design and protocol

This scoping review followed the Preferred Reporting Items for Systematic Reviews and Meta-analysis extension for Scoping Reviews^21,22^ and included published, peer-reviewed articles with original data. A review protocol was developed based on the Preferred Reporting Items for Systematic Reviews and Meta-analysis-Protocols Guidelines.^21,23^ The protocol was not registered online because the primary aim of this work was exploratory, focusing on mapping existing evidence rather than hypothesis testing.

### Eligibility criteria

Studies were considered eligible for inclusion if they (1) were conducted in humans with a transtibial or transfemoral socket prosthesis; (2) compared at least 2 of the 3 shape capture and/or shape design techniques (manual, hybrid, digital) for the prosthetic socket; and (3) measured outcome in (cost-)effectiveness of shape capture and shape design techniques. Studies were excluded if (1) they did not use original data; (2) they included only pediatric patients; (3) they were a conference abstract, conference proceeding, letter to the editor, or textbook; (4) the full text was not available; and (5) the language was non-English and non-Dutch.

### Information sources

A systematic search for studies relating to the comparison of (cost-)effectiveness of manual, hybrid, and digital shape capture and shape design methods for transtibial and transfemoral prosthetic sockets was performed in the databases MEDLINE (through PubMed), Embase (through Ovid), Web of Science, Cochrane, and CINAHL (through EBSCO). Searches were conducted from database inception to September 20, 2023, without date restrictions, to capture both foundational and recent developments in socket shape capture and design methods.

### Search strategy

A Population, Intervention, Comparator, Outcome-based search strategy was developed by the first author (P.G.) with support of a medical librarian. To conduct a complete and inclusive search, the comparator and outcome components were omitted. This approach was chosen because relevant studies in this field often vary in their terminology and may not explicitly report comparator groups or outcome measures in titles and abstracts. Including these elements could, therefore, have unintentionally excluded relevant studies.

The search query for PubMed is given in Table 1. The full search queries for each database can be found in Supplementary Digital Content 1, http://links.lww.com/POI/A360. No search limits were used. Gray literature was excluded to maintain methodological rigor and comparability, focusing instead on peer-reviewed studies to ensure transparency and reproducibility.

**Table 1.**
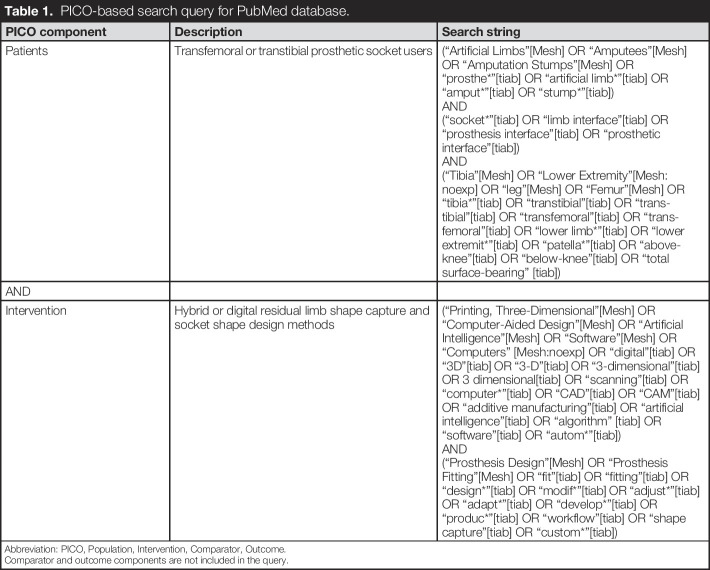
PICO-based search query for PubMed database.

PICO component	Description	Search string
Patients	Transfemoral or transtibial prosthetic socket users	(“Artificial Limbs”[Mesh] OR “Amputees”[Mesh] OR “Amputation Stumps”[Mesh] OR “prosthe*”[tiab] OR “artificial limb*”[tiab] OR “amput*”[tiab] OR “stump*”[tiab]) AND (“socket*”[tiab] OR “limb interface”[tiab] OR “prosthesis interface”[tiab] OR “prosthetic interface”[tiab]) AND (“Tibia”[Mesh] OR “Lower Extremity”[Mesh:noexp] OR “leg”[Mesh] OR “Femur”[Mesh] OR “tibia*”[tiab] OR “transtibial”[tiab] OR “trans-tibial”[tiab] OR “transfemoral”[tiab] OR “trans-femoral”[tiab] OR “lower limb*”[tiab] OR “lower extremit*”[tiab] OR “patella*”[tiab] OR “above-knee”[tiab] OR “below-knee”[tiab] OR “total surface-bearing” [tiab])
AND		
Intervention	Hybrid or digital residual limb shape capture and socket shape design methods	(“Printing, Three-Dimensional”[Mesh] OR “Computer-Aided Design”[Mesh] OR “Artificial Intelligence”[Mesh] OR “Software”[Mesh] OR “Computers” [Mesh:noexp] OR “digital”[tiab] OR “3D”[tiab] OR “3-D”[tiab] OR “3-dimensional”[tiab] OR 3 dimensional[tiab] OR “scanning”[tiab] OR “computer*”[tiab] OR “CAD”[tiab] OR “CAM”[tiab] OR “additive manufacturing”[tiab] OR “artificial intelligence”[tiab] OR “algorithm” [tiab] OR “software”[tiab] OR “autom*”[tiab]) AND (“Prosthesis Design”[Mesh] OR “Prosthesis Fitting”[Mesh] OR “fit”[tiab] OR “fitting”[tiab] OR “design*”[tiab] OR “modif*”[tiab] OR “adjust*”[tiab] OR “adapt*”[tiab] OR “develop*”[tiab] OR “produc*”[tiab] OR “workflow”[tiab] OR “shape capture”[tiab] OR “custom*”[tiab])

Abbreviation: PICO, Population, Intervention, Comparator, Outcome.

Comparator and outcome components are not included in the query.

### Selection process

Deduplication was performed in Endnote. All studies identified by the initial search were screened on title and abstract to check for eligibility based on the inclusion and exclusion criteria. Full text was obtained when studies fit the inclusion criteria or when abstracts were not available. Eligible studies were subsequently assessed on full text and included for analysis based on the inclusion criteria. Screening on title and abstract, and full text, was performed by 2 authors (P.G. and I.P.J.S.). The screening process was conducted within Population, Intervention, Comparator, Outcome Portal, which is a web-based systematic review management tool.^24^

### Data extraction

One reviewer (P.G.) conducted data extraction using a data extraction form customized for this scoping review. The following items were extracted from each eligible study: (1) study characteristics (authors, publication year, study design, study duration); (2) participant characteristics (sample size, subgroups, age, gender, amputation level); (3) intervention and comparator details (type of shape capture and/or socket shape design method); and (4) outcome measures (primary and secondary). Missing data were described as “not reported.” Aspects that were not clearly described or could not be adequately interpreted were described as “not specified.”

### Quality assessment

The methodological quality of the included studies was evaluated by one author (P.G.) using the Effective Public Health Practice Project (EPHPP) Quality Assessment Tool for Quantitative Studies.^25,26^ Given the scoping nature of this review, this approach was deemed appropriate for an exploratory evidence mapping rather than a meta-analytic synthesis. The EPHPP tool was selected because of anticipated variations in study designs and its recognized reliability and validity in critically appraising study methodology.^27,28^ The EPHPP tool assesses 6 methodological components: (1) selection bias, (2) study design, (3) confounders, (4) blinding, (5) data collection methods, and (6) withdrawals and drop-outs. Each component was assigned a rating of “strong,” “moderate,” or “weak” based on established criteria outlined in the EPHPP Dictionary.^24^ The overall quality rating (i.e., global rating) for each study was derived from these individual component ratings. If no component was rated as “weak,” the study received a global rating of “strong”; a single “weak” component resulted in “moderate,” and 2 or more “weak” components led to a global rating of “weak.”

### Synthesis of results

The findings of this review were presented descriptively, supported by a visual representation. Studies were grouped based on the comparison between the manual, hybrid, and digital methods. Socket shape design techniques were divided into 3 levels of standardization: not standardized, semi-standardized, and fully standardized. Measurement constructs were reported with their corresponding measurement instruments. Measurement constructs for effectiveness were categorized based on the International Classification of Functioning, Disability and Health (ICF) domains. The ICF functions as a framework and classification system that provides the foundation for developing tools to measure individual functioning or for aligning these measurement tools with its categories. The ICF uses the main domains “Body functions,” “Body structures,” “Activities and participation,” and “Environmental factors.”^29^ For the sake of conciseness, the ICF categories “Body functions” and “Body structures” were merged in this review. Outcome measures that did not align with any ICF domain were grouped under “Not defined or covered in the ICF.”

The classification of measurement constructs and instruments into ICF domains was based on the COMPASS guidelines,^30^ categories within the instrument itself,^31,32^ and previous studies that applied the ICF linking rules.^33,34^ These linking rules ensure that health information can be accurately linked to the ICF framework, facilitating a consistent and transparent categorization approach.^35^ Although the direct application of these rules was beyond this review’s scope, relying on studies that already employed these rules allowed for robust mapping of outcomes to ICF domains, enhancing the comparability and validity of the findings.

The categorization was performed by one author (P.G.) based on these sources. Because some measurement constructs encompassed multiple functional components and did not clearly correspond to a single ICF domain, classification was based on the predominant aspect described in the study.

## Results

### Search results

**Figure 1.**
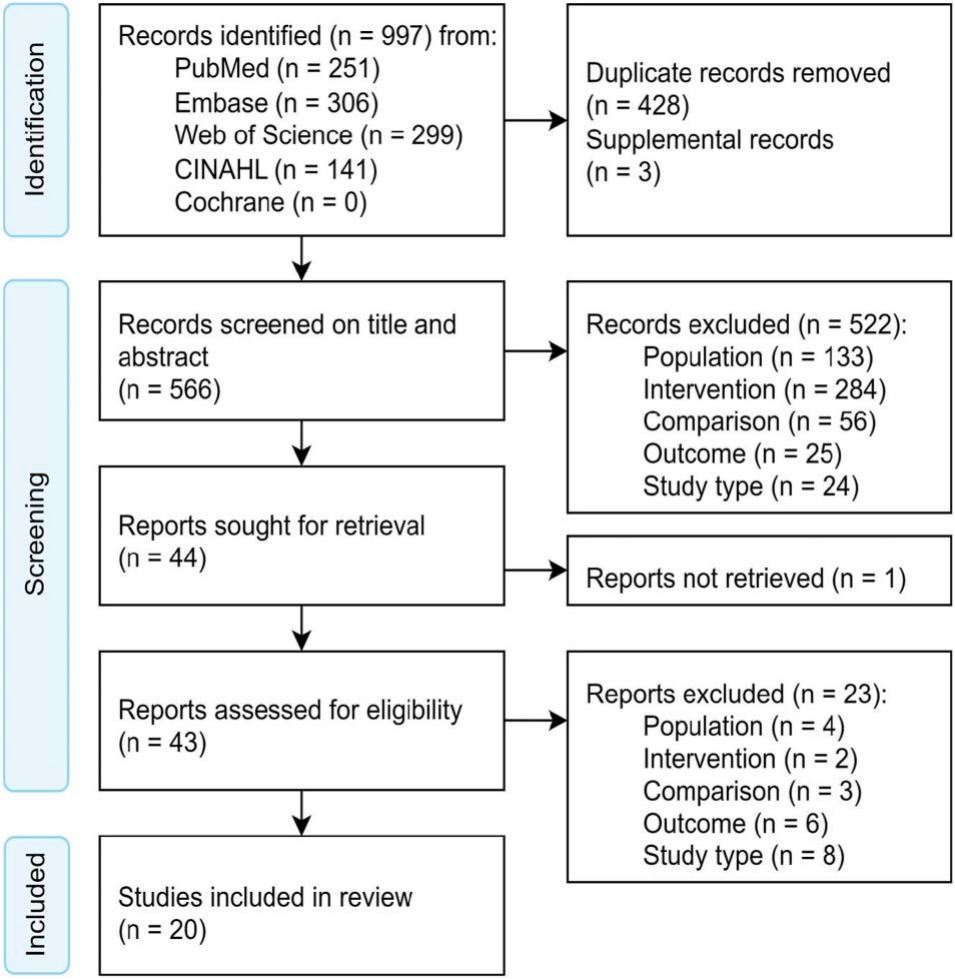
PRISMA flow diagram for search method. PRISMA, preferred reporting items for systematic reviews and meta-analysis.

The search across databases yielded 997 articles of which 566 articles remained after removing duplicates. Twenty articles were included after the application of the inclusion criteria, as illustrated in Figure 1.

### Study characteristics

The selected studies assessed a total of 497 patients, aged 5 to 81 years old. Most studies evaluated transtibial prostheses (n = 16, 431 patients),^36–51^ whereas 4 studies evaluated transfemoral prostheses (66 patients).^52–55^ Seventeen studies used a within-subject^36–44,47–52,54,55^ and 3 studies a between-subject^45,46,53^ study design. The sample size ranged from 1 to 90 participants. The duration of the studies ranged from a single session to 1 year and was not reported in 4 reports.^41,44,47,55^ The geographic distribution of the studies was diverse, including research conducted in Canada (n = 8),^38,39,41,44,45,48,50,54^ the United States (n = 1),^49^ China (n = 2),^51,55^ India (n = 2),^52^ Sweden (n = 2),^36,37^ Turkey (n = 2),^46,53^ Taiwan (n = 1),^43^ Vietnam (n = 1),^42^ and Uganda, Cambodia, and Tanzania (n = 1).^40^ The studies were published between 1985 and 2023. Table 2 provides an overview of the study characteristics.

**Table 2.**
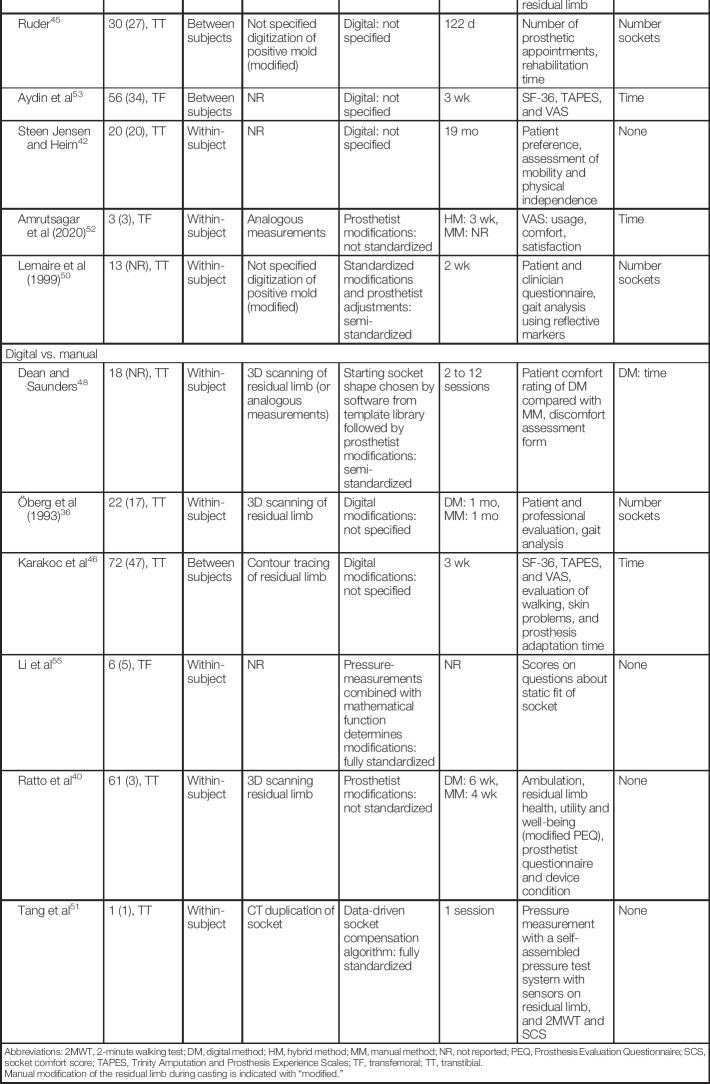
Overview of study characteristics.

Authors (y)	N (males), amputation level	Study design	Shape capture method	Socket shape design method: level of standardization	Trial duration	Effectiveness measure	Cost-effectiveness measure
Hybrid vs. manual							
Köhler et al^37^	8 (NR), TT	Within-subject	Contour tracing of negative mold	Standardized modifications and prosthetist adjustments: semi-standardized	HM: 8 wk, MM: 8 wk	VAS: comfort, pressure and pain	Number socket modifications
Holden and Fernie^38^	10 (NR), TT	Within-subject	Analogous measurements	Standardized modifications and prosthetist adjustments: semi-standardized	20 min, optional continuation	Patient preference and prosthetist evaluation	Time
Topper and Fernie^39^	48 (41), TT	Within-subject	Analogous measurements	Starting socket shape chosen by software from template library followed by prosthetist modifications: semi-standardized	1 session	Patient preference	Number sockets
Topper and Fernie^41^	20 (16), TT	Within-subject	Analogous measurements	Starting socket shape chosen by software from template library followed by prosthetist modifications: semi-standardized	NR	Patient preference	None
Engsberg et al^44^	15 (NR), TT	Within-subject	3D scanning of residual limb	Standardized modifications and prosthetist adjustments: semi-standardized	NR	Patient preference	None
Nayak et al^47^	1 (NR), TT	Within-subject	3D scanning of positive mold (modified)	Postprocessing of residual limb model: not standardized	NR	Socket fit using dimensional evaluation of socket	Time
Torres-Moreno et al^54^	1 (NR), TF	Within-subject	Analogous measurements	Starting socket shape chosen by software from template library followed by prosthetist modifications: semi-standardized	2 trials, HM: 2-mo follow-up	Pressure marks and patient evaluation, cross-sectional area comparison	None
Houston et al^49^	90 (80), TT	Within-subject	Not specified digitization of negative mold or analogous measurements	Starting socket shape chosen by software from template library followed by prosthetist modifications: semi-standardized	HM: 1 mo, MM: 0.25 to 12 y	Rating scale for fit, comfort, function, cosmesis, and weight, overall satisfaction	HM: time, number sockets
Tzeng et al^43^	2 (2), TT	Within-subject	3D scanning of positive mold (modified)	Prosthetist modifications: not standardized	NR for trial. HM: follow-up 1 y	Patient judgement on socket fit, residual limb-socket interface pressure measurement with sensors on residual limb	None
Ruder^45^	30 (27), TT	Between subjects	Not specified digitization of positive mold (modified)	Digital: not specified	122 d	Number of prosthetic appointments, rehabilitation time	Number sockets
Aydin et al^53^	56 (34), TF	Between subjects	NR	Digital: not specified	3 wk	SF-36, TAPES, and VAS	Time
Steen Jensen and Heim^42^	20 (20), TT	Within-subject	NR	Digital: not specified	19 mo	Patient preference, assessment of mobility and physical independence	None
Amrutsagar et al (2020)^52^	3 (3), TF	Within-subject	Analogous measurements	Prosthetist modifications: not standardized	HM: 3 wk, MM: NR	VAS: usage, comfort, satisfaction	Time
Lemaire et al (1999)^50^	13 (NR), TT	Within-subject	Not specified digitization of positive mold (modified)	Standardized modifications and prosthetist adjustments: semi-standardized	2 wk	Patient and clinician questionnaire, gait analysis using reflective markers	Number sockets
Digital vs. manual							
Dean and Saunders^48^	18 (NR), TT	Within-subject	3D scanning of residual limb (or analogous measurements)	Starting socket shape chosen by software from template library followed by prosthetist modifications: semi-standardized	2 to 12 sessions	Patient comfort rating of DM compared with MM, discomfort assessment form	DM: time
Öberg et al (1993)^36^	22 (17), TT	Within-subject	3D scanning of residual limb	Digital modifications: not specified	DM: 1 mo, MM: 1 mo	Patient and professional evaluation, gait analysis	Number sockets
Karakoc et al^46^	72 (47), TT	Between subjects	Contour tracing of residual limb	Digital modifications: not specified	3 wk	SF-36, TAPES, and VAS, evaluation of walking, skin problems, and prosthesis adaptation time	Time
Li et al^55^	6 (5), TF	Within-subject	NR	Pressure-measurements combined with mathematical function determines modifications: fully standardized	NR	Scores on questions about static fit of socket	None
Ratto et al^40^	61 (3), TT	Within-subject	3D scanning residual limb	Prosthetist modifications: not standardized	DM: 6 wk, MM: 4 wk	Ambulation, residual limb health, utility and well-being (modified PEQ), prosthetist questionnaire and device condition	None
Tang et al^51^	1 (1), TT	Within-subject	CT duplication of socket	Data-driven socket compensation algorithm: fully standardized	1 session	Pressure measurement with a self-assembled pressure test system with sensors on residual limb, and 2MWT and SCS	None

Abbreviations: 2MWT, 2-minute walking test; DM, digital method; HM, hybrid method; MM, manual method; NR, not reported; PEQ, Prosthesis Evaluation Questionnaire; SCS, socket comfort score; TAPES, Trinity Amputation and Prosthesis Experience Scales; TF, transfemoral; TT, transtibial.

Manual modification of the residual limb during casting is indicated with “modified.”

The comparison between manual and hybrid methods was conducted in 14 studies (11 transtibial, 3 transfemoral).^37–39,41–45,47,49,50,52–54^ The comparison between manual and digital methods was conducted in 6 studies (5 transtibial, 1 transfemoral).^36,40,46,48,51,55^ The study by Dean and Saunders^48^ provided 2 options for shape capture, namely 3D scanning or analogous measurements but was classified here as a digital method. Li et al.^55^ focused solely on evaluating the socket shape design technique, without assessing the shape capture technique.^50^ No studies comparing hybrid and digital methods were identified, indicating that direct comparative data between these increasingly applied techniques are currently unavailable.

### Quality assessment

Quality assessment rated 18 studies to have “low,” 2 studies to have “moderate,” and none to have “high” quality. The main reasons for the low or moderate ratings were related to study design limitations, including the absence of randomization, lack of blinding, small sample sizes, and limited control for confounding variables. Most studies used observational or quasi-experimental designs without comparator standardization, which inherently constrained methodological rigor. The results of the quality assessment are summarized in Table 3, with detailed assessments available in Supplementary Digital Content 2, http://links.lww.com/POI/A361.

**Table 3.**
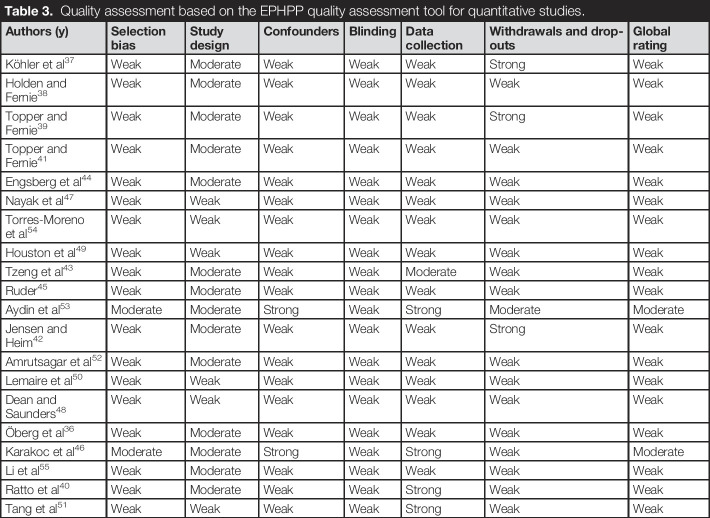
Quality assessment based on the EPHPP quality assessment tool for quantitative studies.

Authors (y)	Selection bias	Study design	Confounders	Blinding	Data collection	Withdrawals and drop-outs	Global rating
Köhler et al^37^	Weak	Moderate	Weak	Weak	Weak	Strong	Weak
Holden and Fernie^38^	Weak	Moderate	Weak	Weak	Weak	Weak	Weak
Topper and Fernie^39^	Weak	Moderate	Weak	Weak	Weak	Strong	Weak
Topper and Fernie^41^	Weak	Moderate	Weak	Weak	Weak	Weak	Weak
Engsberg et al^44^	Weak	Moderate	Weak	Weak	Weak	Weak	Weak
Nayak et al^47^	Weak	Weak	Weak	Weak	Weak	Weak	Weak
Torres-Moreno et al^54^	Weak	Weak	Weak	Weak	Weak	Weak	Weak
Houston et al^49^	Weak	Weak	Weak	Weak	Weak	Weak	Weak
Tzeng et al^43^	Weak	Moderate	Weak	Weak	Moderate	Weak	Weak
Ruder^45^	Weak	Moderate	Weak	Weak	Weak	Weak	Weak
Aydin et al^53^	Moderate	Moderate	Strong	Weak	Strong	Moderate	Moderate
Jensen and Heim^42^	Weak	Moderate	Weak	Weak	Weak	Strong	Weak
Amrutsagar et al^52^	Weak	Moderate	Weak	Weak	Weak	Weak	Weak
Lemaire et al^50^	Weak	Weak	Weak	Weak	Weak	Weak	Weak
Dean and Saunders^48^	Weak	Weak	Weak	Weak	Weak	Weak	Weak
Öberg et al^36^	Weak	Moderate	Weak	Weak	Weak	Weak	Weak
Karakoc et al^46^	Moderate	Moderate	Strong	Weak	Strong	Weak	Moderate
Li et al^55^	Weak	Moderate	Weak	Weak	Weak	Weak	Weak
Ratto et al^40^	Weak	Moderate	Weak	Weak	Strong	Weak	Weak
Tang et al^51^	Weak	Weak	Weak	Weak	Strong	Weak	Weak

### Shape capture and socket shape design techniques

Details of the manual socket were documented in 15 studies. The manual socket was made using plaster or fiberglass casting (n = 14) or vacuum casting (n = 1) followed by manual modifications made by a prosthetist. In one study, the manual socket consisted of a duplicate of the socket before a quantitative compensation method was applied.^50^ Four studies did not report any details about the manual socket used as the comparison arm.

**Figure 2.**
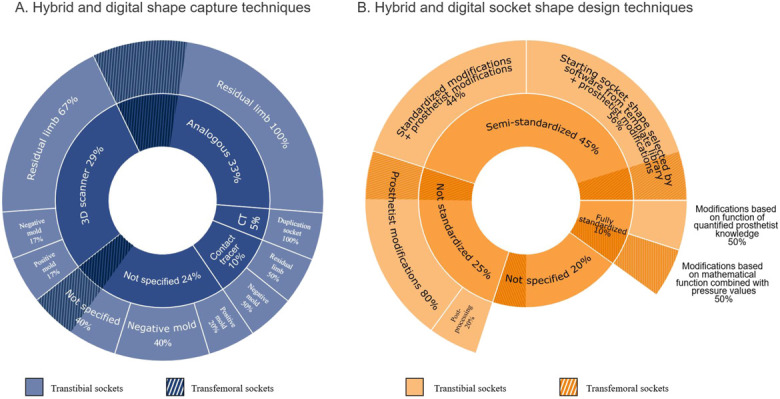
Overview of classification of hybrid and digital residual limb shape capture and socket shape design techniques. (a) The inner ring represents major shape capture categories; the outer ring specifies the particular aspects or elements that are captured within each method. (b) The inner ring categorizes major socket shape design categories based on different levels of standardization; the outer ring specifies design techniques employed within each standardization level. Percentages reflect the distribution of studies across these categories. Unstriped segments represent studies evaluating transtibial sockets, whereas striped segments indicate those focusing on transfemoral sockets. CT, computed tomography.

Among the hybrid and digital methods, shape capture techniques could be categorized into analogous (n = 7),^38,39,41,48,49,52,54^ 3D scanner (n = 5),^36,40,43,44,47^ contact tracer (n = 2),^37,46^ or computed tomography (n = 1).^51^ In 5 studies, this was not specified or reported.^42,45,49,50,53^ Analogous measurement-based methods used tape^52^ and caliper^39,41,48,49,52^ measurements, casting jigs,^39,41^ or undefined analogous measurements.^38,54^ Scanner-based methods digitized either a negative^47^ or positive^43,44^ mold of the residual limb, or the residual limb itself^36,40,48^ using laser or structured light. Tracer-based methods digitized a negative plaster cast by moving a probe across the surface of the residual limb^46^ or along the inner surface of a negative plaster cast.^37^ An overview is provided in Figure 2(a). This figure also illustrates that the shape capture techniques for hybrid and digital methods mainly focus on transtibial sockets (n = 16). For transfemoral sockets, the techniques used were either analogous (n = 2)^52,54^ or not specified (n = 1).^53^

Socket shape design techniques among the hybrid and digital methods were categorized into not standardized (n = 5),^40,43,46,47,52^ semi-standardized (n = 9),^37–39,41,44,48–50,54^ and fully standardized (n = 2).^51,55^ Four studies did not specify the socket shape design technique.^36,42,45,53^ The resulting model was a 3D model of the residual limb (i.e., a positive mold to manufacture the socket around) in most studies (n = 14),^36–39,41,43–46,48–50,52,54^ or a 3D model of the socket (n = 3).^40,47,51^ Three studies did not report this.^42,53,55^ This is visualized in Figure 2(b). Although there were few studies available, the shape design techniques for transfemoral sockets varied, including nonstandardized (n = 1),^52^ semi-standardized (n = 1),^54^ fully standardized (n = 1),^55^ and not specified (n = 1).^53^ Across these categories, several studies did not report key methodological details, including the specific shape capture technique, socket shape design approach, or level of standardization. As a result, information was incomplete for some comparisons presented in Figure 2.

### Measurement constructs and instruments used

Table 4 offers an overview of the measurement constructs and corresponding instruments used to assess various forms of effectiveness (n = 20) and cost-effectiveness (n = 10) across the included studies. The most frequently assessed constructs were socket fit (n = 7), socket comfort (n = 6), pain (n = 5), pressure (n = 5), and patient preference (n = 5). Common instruments included the visual analog scale (VAS) (n = 4) and direct patient comparisons between sockets (n = 4). Within the ICF framework, 9 studies addressed Body Functions and Structures and 7 addressed Activities and Participation, whereas none reported measures in the Environmental Factors domain. All 20 studies also included constructs outside the ICF framework. Cost-related outcomes were primarily captured through indicators such as measurement procedure time (n = 6) and the number of socket attempts required for a successful fit (n = 4). Two studies solely reported the time and/or socket attempts required for developing a socket using a hybrid method, without comparison to manual methods.^48,49^

**Table 4.**
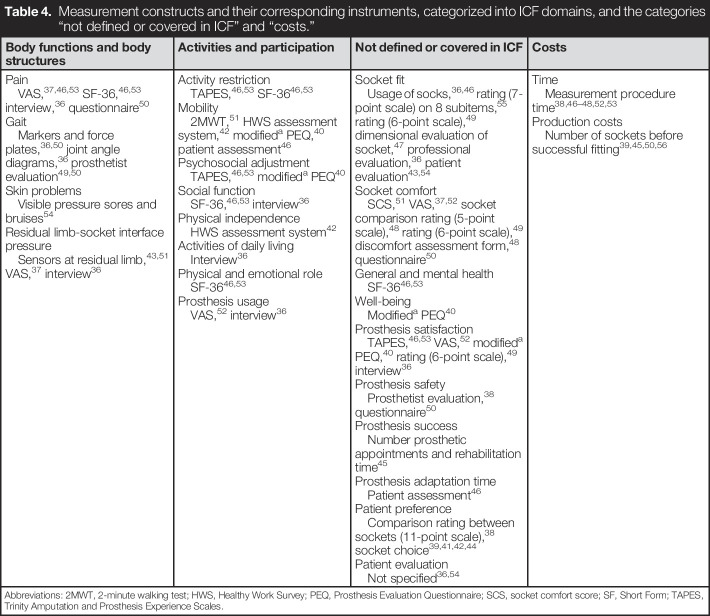
Measurement constructs and their corresponding instruments, categorized into ICF domains, and the categories “not defined or covered in ICF” and “costs.”

Body functions and body structures	Activities and participation	Not defined or covered in ICF	Costs
Pain VAS,^37,46,53^ SF-36,^46,53^ interview,^36^ questionnaire^50^ Gait Markers and force plates,^36,50^ joint angle diagrams,^36^ prosthetist evaluation^49,50^ Skin problems Visible pressure sores and bruises^54^ Residual limb-socket interface pressure Sensors at residual limb,^43,51^ VAS,^37^ interview^36^	Activity restriction TAPES,^46,53^ SF-36^46,53^ Mobility 2MWT,^51^ HWS assessment system,^42^ modified^a^ PEQ,^40^ patient assessment^46^ Psychosocial adjustment TAPES,^46,53^ modified^a^ PEQ^40^ Social function SF-36,^46,53^ interview^36^ Physical independence HWS assessment system^42^ Activities of daily living Interview^36^ Physical and emotional role SF-36^46,53^ Prosthesis usage VAS,^52^ interview^36^	Socket fit Usage of socks,^36,46^ rating (7-point scale) on 8 subitems,^55^ rating (6-point scale),^49^ dimensional evaluation of socket,^47^ professional evaluation,^36^ patient evaluation^43,54^ Socket comfort SCS,^51^ VAS,^37,52^ socket comparison rating (5-point scale),^48^ rating (6-point scale),^49^ discomfort assessment form,^48^ questionnaire^50^ General and mental health SF-36^46,53^ Well-being Modified^a^ PEQ^40^ Prosthesis satisfaction TAPES,^46,53^ VAS,^52^ modified^a^ PEQ,^40^ rating (6-point scale),^49^ interview^36^ Prosthesis safety Prosthetist evaluation,^38^ questionnaire^50^ Prosthesis success Number prosthetic appointments and rehabilitation time^45^ Prosthesis adaptation time Patient assessment^46^ Patient preference Comparison rating between sockets (11-point scale),^38^ socket choice^39,41,42,44^ Patient evaluation Not specified^36,54^	Time Measurement procedure time^38,46–48,52,53^ Production costs Number of sockets before successful fitting^39,45,50,56^

Abbreviations: 2MWT, 2-minute walking test; HWS, Healthy Work Survey; PEQ, Prosthesis Evaluation Questionnaire; SCS, socket comfort score; SF, Short Form; TAPES, Trinity Amputation and Prosthesis Experience Scales.

### Reported (cost-)effectiveness findings

Across studies, direct comparative evidence on (cost-)effectiveness was limited. Measures of efficiency, such as procedure time and the number of socket iterations, generally favored hybrid or digital methods over manual approaches, although reporting was inconsistent and often descriptive. Outcomes reflecting effectiveness, including socket fit, comfort, and user satisfaction, were assessed using a wide range of subjective and objective methods, such as the Socket Comfort Score, VAS ratings, and professional evaluations. Several studies suggested improved comfort or reduced adjustment needs with digital or hybrid sockets; however, these findings were not consistent across all reports. Overall, the available evidence is heterogeneous and methodologically limited, precluding firm conclusions on relative (cost-)effectiveness.

## Discussion

This scoping review aimed to provide an overview of the existing comparative studies evaluating (cost-)effectiveness of manual, hybrid, and digital shape capture and shape design methods for transtibial and transfemoral prosthetic sockets. In total, 20 studies that addressed this comparison were systematically identified. The key findings of this review are the scarcity of comparative studies between manual, hybrid, and digital methods, and the absence of any comparing hybrid and digital techniques, indicating a clear evidence gap on (cost-)effectiveness. Most studies focused on transtibial sockets and employed heterogeneous shape-capture and design techniques with inconsistent measurement constructs.

### Quality of included studies

The quality assessment of studies in this review indicates methodological weaknesses that compromise the reliability of (cost-)effectiveness analysis for manual, hybrid, and digital shape capture and socket shape design methods. Most studies were assessed as “low” quality, limiting the ability to effectively compare and report on their (cost-)effectiveness. Key issues include selection bias, inadequate blinding, and poor control of confounders, which can partially be attributed to the use of a within-subject study design. Many studies lacked randomization and did not specify inclusion and exclusion criteria, leading to potential biases that affect the generalizability of the findings. Furthermore, the absence of blinding in studies reliant on subjective outcomes, coupled with insufficient control for variables such as patient demographics or prior prosthetic experience, likely distorts intervention effects. Similar observations have been reported by Kooiman et al,^57^ reinforcing the importance of addressing these methodological concerns. In addition, 24% of the included studies failed to specify the shape capture techniques used, and 20% omitted details about the socket shape design techniques employed as interventions. Such omissions in reporting, possibly because of concerns over proprietary information, hinder thorough interpretation and comparison of methods.^58^

### Effectiveness: constructs and instruments

Definitions and measurement of effectiveness varied widely across the included studies, with limited alignment to ICF domains and inconsistent use of validated instruments. Although this broad interpretation reflects the multidimensional nature of prosthetic socket performance, it complicates comparison across studies. Constructs such as socket fit, comfort, pain, and patient preference were frequently assessed, yet often without explicit conceptual definitions or standardized measurement tools, and most relied on subjective, patient-reported outcomes (e.g., questionnaires, interviews, VAS) with few incorporating objective metrics such as digital pressure mapping, limb-volume monitoring, or gait analysis.

We noted frequent instances where the chosen instruments did not perfectly align with the intended constructs. For example, Karakoc et al^46^ and Aydin et al^53^ reported pain and functional outcomes primarily via SF-36 subscores, a generic health-related quality-of-life measure that does not capture socket-specific pain or function in detail. Similarly, Öberg et al^36^ and Karakoc et al^46^ used “sock usage” as a proxy for socket fit, which reflects limb-volume changes but not local fit or pressure distribution. Older studies often used subjective global ratings or unvalidated socket-comparison scales (e.g., Dean & Saunders^48^; Houston et al^49^) to assess comfort, reducing comparability with studies using validated instruments such as the Socket Comfort Score or objective pressure sensors. Finally, metrics such as the number of prosthetic appointments or sockets before successful fitting (Ruder^45^; Topper & Fernie^39^; Lemaire et al^50^) were used as pragmatic proxies for success or cost, but these are influenced by local service organization and do not substitute for formal economic evaluation. These examples illustrate the need for stronger conceptual alignment between constructs and measurement instruments in future studies.

Categorizing outcomes according to ICF domains can further clarify which aspects are being addressed. In this review, outcome measures were linked to ICF domains using previously established mapping frameworks, as described in the Methods section. However, this process was not always straightforward. Several constructs lacked clear definitions or spanned multiple functional components, making classification interpretive. These challenges reflect the complexity of applying the ICF framework to heterogeneous outcome measures in prosthetic socket research. However, few studies explicitly linked their constructs to the ICF framework, and when they did, the focus was mainly on the domains “Body Functions & Structures” and “Activities and Participation,” whereas “Environmental Factors” remained largely unrepresented. This limited scope highlights a need for more comprehensive outcome frameworks that capture the full spectrum of user function and participation relevant to prosthetic socket evaluation.

### Cost-effectiveness: constructs and instruments

The current evidence provides limited insight into the cost-effectiveness of manual, hybrid, and digital shape capture and socket design techniques. Most studies have focused on production time and the number of socket attempts required for a successful fit, metrics that reflect costs rather than comprehensive cost-effectiveness. Factors such as technician experience, particularly with novel methods, may influence these outcomes. Only one study considered both production efficiency and patient satisfaction (fit and comfort), but this involved just 3 patients, limiting generalizability.^52^ Comprehensive cost-effectiveness analyses should account for all relevant costs and outcomes, including health care system, patient-level, and broader societal costs.^20^

Despite these limitations and the generally low methodological quality of the studies, tentative evidence suggests that hybrid and digital methods may reduce production time and the number of socket attempts compared with manual techniques, with possible improvements in efficiency. Likewise, the evidence hints at modest gains in socket fit and patient comfort with digital and hybrid approaches. However, these interpretations are highly tentative because of the scarcity of robust comparative data, inconsistent outcome measures, and limited objective assessments. No studies directly compared hybrid and digital methods, and fully standardized digital approaches remain largely unevaluated. Overall, although early indications point to potential benefits, the current evidence is insufficient to draw definitive conclusions on either cost-effectiveness or effectiveness.

### Shape capture techniques

The included studies employed a variety of techniques for residual limb shape capture, the most common being analogous measurements (e.g., caliper and tape) and 3D scanning of the limb or its positive or negative mold. Manual modifications, such as applying pressure to identify pressure-tolerant regions, were sometimes incorporated to enhance the subsequent digital model, although these methods remain heavily dependent on prosthetist expertise.

This review partially overlaps with the systematic review by Yang et al^17^ who examined shape capture techniques for transtibial sockets but excluded studies that also integrated socket design. Yang et al's review emphasized traditional (largely manual) shape capture approaches, which led to the omission of studies using direct socket modeling or fully digital workflows based on scans of negatives. They further excluded studies using 3D scanning of negative molds, despite its presence in clinical practice. Their search identified only 2 comparative studies on shape capture up to 2018. This selective approach may not encompass all available techniques and emerging innovations. By broadening the scope to include both transtibial and transfemoral prostheses, and socket shape design techniques, the present review complements and expands upon their findings, offering a more comprehensive overview of current and emerging methods.

### Socket shape design techniques

Across the included studies, semi-standardized techniques were the most commonly applied in hybrid and digital approaches, balancing predefined modifications with prosthetist expertise. Not standardized methods, relying entirely on manual modifications, offered high individualization but limited reproducibility. Fully standardized techniques were rarely evaluated, and their clinical and economic impact remains largely unknown. The reliance on prosthetist skill in semi-standardized methods may introduce variability in outcomes and limit consistency across practitioners, highlighting the potential value of further standardization.

It was anticipated that some studies would evaluate innovations incorporating artificial intelligence^59^ or statistical shape models^60^; however, no evidence was found assessing the additional benefits of such methods. As these technologies become increasingly prevalent, rigorous studies are needed to determine their (cost-)effectiveness before widespread adoption in clinical practice.

### Limitations

Two limitations should be acknowledged in this study. First, the quality assessment was performed by a single reviewer, which may introduce some risk of bias. However, this risk is considered limited, as most studies were rated as low quality because of clear design-related shortcomings, such as nonrandomized or uncontrolled designs and lack of blinding, rather than subjective interpretation of minor criteria. Therefore, a second reviewer would be unlikely to alter the overall quality classification. The decision to proceed with a single-reviewer appraisal was consistent with the exploratory nature of this scoping review, which prioritized comprehensive mapping of evidence over formal critical synthesis. Second, the classification of measurement outcomes and instruments into ICF domains did not employ the ICF linking rules, as applying these rules was considered too extensive and beyond the scope of this review. Instead, the classification was based on interpretations from other studies that had previously applied these linking rules, which introduces a potential limitation in the accuracy and consistency of categorization. Nevertheless, classification according to ICF categories in this scoping review provides a structured framework for assessing various dimensions of health and functioning and promoting comparability across studies.

### Future directions

To improve comparability and cumulative evidence, future studies should adopt a core set of standardized patient characteristics, testing protocols, and validated outcome measures aligned with ICF domains. Combining subjective and objective tools, such as gait analysis, pressure mapping, and residual-limb volume assessment, would enable more reliable evaluation of socket performance. Transparent descriptions of shape capture and design methods are essential to improve reproducibility. At the same time, robust multicenter trials and comprehensive cost-effectiveness analyses that include both health care system and patient-level costs are needed to guide evidence-based clinical implementation of emerging hybrid and digital technologies.

## Conclusion

This review demonstrates that current evidence on the (cost-)effectiveness of manual, hybrid, and digital socket design methods remains weak and fragmented. Early findings cautiously suggest that hybrid and digital techniques may enhance efficiency and user comfort compared with manual approaches, but methodological limitations prevent firm conclusions. Strengthening study quality, consistency of outcome measures, and economic evaluation will be crucial to determine the real clinical value of these evolving technologies.

## Declaration of conflicting interest

## Supplementary Material
