# Supplementary material for: Comparisons of (cost-)effectiveness of manual, hybrid, and digital shape capture and shape design techniques for transtibial and transfemoral prosthetic sockets: A scoping review
Source: Prosthet Orthot Int. 2026 Mar 11;50(4):491–503. doi: 10.1097/PXR.0000000000000531 (PMC13440530; doi:10.1097/PXR.0000000000000531)
Supplement: Supplementary file 1 [file poi-50-491-s001.docx]

**Supplemental Digital Content 1**

**Pubmed
September 20, 2023
Results: 251**

(”Artificial Limbs”[Mesh] OR ”Amputees”[Mesh] OR ”Amputation Stumps”[Mesh] OR ”prosthe*”[tiab] OR ”artificial limb*”[tiab] OR ”amput*”[tiab] OR ”stump*”[tiab])

AND

(”socket*”[tiab] OR ”limb interface”[tiab] OR ”prosthesis interface”[tiab] OR ”prosthetic interface”[tiab]) AND

(”Tibia”[Mesh] OR ”Lower Extremity”[Mesh:noexp] OR ”leg”[Mesh] OR ”Femur”[Mesh] OR ”tibia*”[tiab] OR ”transtibial”[tiab] OR ”trans-tibial”[tiab] OR ”transfemoral”[tiab] OR ”trans-femoral”[tiab] OR ”lower limb*”[tiab] OR ”lower extremit*”[tiab] OR ”patella*”[tiab] OR ”above-knee”[tiab] OR ”below-knee”[tiab] OR ”total surface-bearing” [tiab])

AND

(”Printing, Three-Dimensional”[Mesh] OR ”Computer-Aided Design”[Mesh] OR ”Artificial Intelligence”[Mesh] OR ”Software”[Mesh] OR ”Computers” [Mesh:noexp] OR ”digital”[tiab] OR ”3D”[tiab] OR ”3-D”[tiab] OR ”3-dimensional”[tiab] OR three dimensional[tiab] OR ”scanning”[tiab] OR ”computer*”[tiab] OR ”CAD”[tiab] OR ”CAM”[tiab] OR ”additive manufacturing”[tiab] OR ”artificial intelligence”[tiab] OR ”algorithm” [tiab] OR ”software”[tiab] OR ”autom*”[tiab])

AND

(”Prosthesis Design”[Mesh] OR ”Prosthesis Fitting”[Mesh] OR ”fit”[tiab] OR ”fitting”[tiab] OR ”design*”[tiab] OR ”modif*”[tiab] OR ”adjust*”[tiab] OR ”adapt*”[tiab] OR ”develop*”[tiab] OR ”produc*”[tiab] OR ”workflow”[tiab] OR ”shape capture”[tiab] OR ”custom*”[tiab])

**Embase
September 20, 2023 Results: 306**

(exp ”limb prosthesis”/ or exp ”amputee”/ or exp ”amputation stump”/ or prosthe*.ti,ab. or ”artificial limb*”.ti,ab. or amput*.ti,ab. or stump*.ti,ab.)

and

(socket* or ”limb interface” or ”prosthesis interface” or ”prosthetic interface”).ti,ab. and

(exp ”tibia”/ or ”lower limb”/ or exp ”leg”/ or exp ”femur”/ or ”tibia*”.ti,ab. or ”transtibial”.ti,ab. or ”trans-tibial”.ti,ab. or ”transfemoral”.ti,ab. or ”trans-femoral”.ti,ab. or ”lower limb*”.ti,ab. or ”lower extremit*”.ti,ab. or ”patella*”.ti,ab. or ”above-knee”.ti,ab. or ”below-knee”.ti,ab. or ”total surface-bearing”.ti,ab.)

and

(exp ”computer aided design”/ or exp ”three dimensional printing”/ or exp ”artificial intelligence”/ or exp ”software”/ or ”computer”/ or ”digital”.ti,ab. or ”3D”.ti,ab. or ”3-D”.ti,ab. or ”3-dimensional”.ti,ab. or ”three dimensional”.ti,ab. or ”scanning”.ti,ab. or ”computer*”.ti,ab. or ”CAD”.ti,ab. or ”CAM”.ti,ab. or ”additive manufacturing”.ti,ab. or ”artificial intelligence”.ti,ab. or ”algorithm”.ti,ab. or ”software”.ti,ab. or autom*.ti,ab.)

and

(exp ”prosthesis design”/ or exp ”prosthetic procedure”/ or ”fit”.ti,ab. or ”fitting”.ti,ab. or ”design*”.ti,ab. or ”modif*”.ti,ab. or ”adjust*”.ti,ab. or ”adapt*”.ti,ab. or ”develop*”.ti,ab. or ”produc*”.ti,ab. or ”workflow”.ti,ab. or ”shape capture”.ti,ab. or ”custom*”.ti,ab.)

**Web of Science
September 20, 2023 Results: 299**

(TS=”Artificial Limbs” OR TS=Amputees OR TS=”Amputation Stumps” OR (TI=prosthe* OR AB=prosthe*) OR (TI=”artificial limb*” OR AB=”artificial limb*”) OR (TI=amput* OR AB=amput*) OR (TI=stump* OR AB=stump*))

AND

((TI=”socket*” OR AB=”socket*”) OR (TI=”limb interface” OR AB=”limb interface”) OR (TI=”prosthesis interface” OR AB=”prosthesis interface”) OR (TI=”prosthetic interface” OR AB=”prosthetic interface”))

AND

(TS=Tibia OR TS=”Lower Extremity” OR TS=leg OR TS=Femur OR (TI=tibia* OR AB=tibia*) OR (TI=transtibial OR AB=transtibial) OR (TI=trans-tibial OR AB=trans-tibial) OR (TI=transfemoral OR AB=transfemoral) OR (TI=trans-femoral OR AB=trans-femoral) OR (TI=”lower limb*” OR AB=”lower limb*”) OR (TI=”lower extremit*” OR AB=”lower extremit*”) OR (TI=patella* OR AB=patella*) OR (TI=above-knee OR AB=above-knee) OR (TI=below-knee OR AB=below-knee) OR (TI=”total surface-bearing” OR AB=”total surface-bearing”))

AND

(TS=”Printing, Three-Dimensional” OR TS=”Computer-Aided Design” OR TS=”Artificial Intelligence” OR TS=Software OR TS=Computers OR (TI=digital OR AB=digital) OR (TI=3D OR AB=3D) OR (TI=3-D OR AB=3-D) OR (TI=3-dimensional OR AB=3-dimensional) OR (TI=”three dimensional” OR AB=”three dimensional”) OR (TI=scanning OR AB=scanning) OR (TI=computer* OR AB=computer*) OR (TI=CAD OR AB=CAD) OR (TI=CAM OR AB=CAM) OR (TI=”additive manufacturing” OR AB=”additive manufacturing”) OR (TI=”artificial intelligence” OR AB=”artificial intelligence”) OR (TI=algorithm OR AB=algorithm) OR (TI=software OR AB=software) OR (TI=autom* OR AB=autom*))

AND

(TS=”Prosthesis Design” OR TS=”Prosthesis Fitting” OR (TI=fit OR AB=fit) OR (TI=fitting OR AB=fitting) OR (TI=design* OR AB=design*) OR (TI=modif* OR AB=modif*) OR (TI=adjust* OR AB=adjust*) OR (TI=adapt* OR AB=adapt*) OR (TI=develop* OR AB=develop*) OR (TI=produc* OR AB=produc*) OR (TI=workflow OR AB=workflow) OR (TI=”shape capture” OR AB=”shape capture”) OR (TI=custom* OR AB=custom*))

**CINAHL
September 20, 2023 Results: 141**

((MH ”Artificial Limbs+”) OR (MH Amputees+) OR (MH ”Amputation Stumps+”) OR (TI prosthe* OR AB prosthe*) OR (TI ”artificial limb*” OR AB ”artificial limb*”) OR (TI amput* OR AB amput*) OR (TI stump* OR AB stump*))

AND

((TI socket* OR AB socket*) OR (TI ”limb interface” OR AB ”limb interface”) OR (TI ”prosthesis interface” OR AB ”prosthesis interface”) OR (TI ”prosthetic interface” OR AB ”prosthetic interface”))

AND

((MH Tibia+) OR (MH ”Lower Extremity”) OR (MH leg+) OR (MH Femur+) OR (TI tibia* OR AB tibia*) OR (TI transtibial OR AB transtibial) OR (TI trans-tibial OR AB trans-tibial) OR (TI transfemoral OR AB transfemoral) OR (TI trans-femoral OR AB trans-femoral) OR (TI ”lower limb*” OR AB ”lower limb*”) OR (TI ”lower extremit*” OR AB ”lower extremit*”) OR (TI patella* OR AB patella*) OR (TI above-knee OR AB above-knee) OR (TI below-knee OR AB below-knee) OR (TI ”total surface-bearing” OR AB ”total surface-bearing”))

AND

(((MH ”Printing, Three-Dimensional+”) OR (MH ”Computer-Aided Design+”) OR (MH ”Artificial Intelligence+”) OR (MH Software+) OR (MH Computers) OR (TI digital OR AB digital) OR (TI 3D OR AB 3D) OR (TI 3-D OR AB 3-D) OR (TI 3-dimensional OR AB 3-dimensional) OR (TI ”three dimensional” OR AB ”three dimensional”) OR (TI scanning OR AB scanning) OR (TI computer* OR AB computer*) OR (TI CAD OR AB CAD) OR (TI CAM OR AB CAM) OR (TI ”additive manufacturing” OR AB ”additive manufacturing”) OR (TI ”artificial intelligence” OR AB ”artificial intelligence”) OR (TI algorithm OR AB algorithm) OR (TI software OR AB software) OR (TI autom* OR AB autom*))

AND

((MH ”Prosthesis Design+”) OR (MH ”Prosthesis Fitting+”) OR (TI fit OR AB fit) OR (TI fitting OR AB fitting) OR (TI design* OR AB design*) OR (TI modif* OR AB modif*) OR (TI adjust* OR AB adjust*) OR (TI adapt* OR AB adapt*) OR (TI develop* OR AB develop*) OR (TI produc* OR AB produc*) OR (TI workflow OR AB workflow) OR (TI ”shape capture” OR AB ”shape capture”) OR (TI custom* OR AB custom*))

**Cochrane,
September 20, 2023 Results: 0**

([mh ”Artificial Limbs”] OR [mh Amputees] OR [mh ”Amputation Stumps”] OR prosthe*:ti,ab OR (”artificial” NEXT limb*):ti,ab OR amput*:ti,ab OR stump*:ti,ab)

AND

(socket*:ti,ab OR ”limb interface”:ti,ab OR ”prosthesis interface”:ti,ab OR ”prosthetic interface”:ti,ab)
AND

([mh Tibia] OR [mh ˆ”Lower Extremity”] OR [mh leg] OR [mh Femur] OR tibia*:ti,ab OR transtibial:ti,ab OR trans-tibial:ti,ab OR transfemoral:ti,ab OR trans-femoral:ti,ab OR (”lower” NEXT limb*):ti,ab OR (”lower” NEXT extremit*):ti,ab OR patella*:ti,ab OR above-knee:ti,ab OR below-knee:ti,ab OR ”total surface-bearing”:ti,ab)

AND

([mh ”Printing, Three-Dimensional”] OR [mh ”Computer-Aided Design”] OR [mh ”Artificial Intelligence”] OR [mh Software] OR [mh

ˆComputers] OR digital:ti,ab OR 3D:ti,ab OR “3 D”:ti,ab OR “3 dimensional”:ti,ab OR ”three dimensional”:ti,ab OR scanning:ti,ab OR computer*:ti,ab OR CAD:ti,ab OR CAM:ti,ab OR ”additive manufacturing”:ti,ab OR ”artificial intelligence”:ti,ab OR algorithm:ti,ab OR software:ti,ab OR autom*:ti,ab)

AND

([mh ”Prosthesis Design”] OR [mh ”Prosthesis Fitting”] OR fit:ti,ab OR fitting:ti,ab OR design*:ti,ab OR modif*:ti,ab OR adjust*:ti,ab OR adapt*:ti,ab OR develop*:ti,ab OR produc*:ti,ab OR workflow:ti,ab OR ”shape capture”:ti,ab OR custom*:ti,ab)
