# Supplementary material for: Comparisons of (cost-)effectiveness of manual, hybrid, and digital shape capture and shape design techniques for transtibial and transfemoral prosthetic sockets: A scoping review
Source: Prosthet Orthot Int. 2026 Mar 11;50(4):491–503. doi: 10.1097/PXR.0000000000000531 (PMC13440530; doi:10.1097/PXR.0000000000000531)
Supplement: Supplementary file 2 [file poi-50-491-s002.docx]

**Supplemental Digital Content 2**

Table B1 shows the detailed quality assessment using the Effective Public Health Practice Project (EPHPP) Quality Assessment Tool for Quantitative Studies.

**Table B1.** Details of quality assessment according to the Effective Public Health Practice Project (EPHPP) Quality Assessment Tool for Quantitative Studies. This tool assesses six methodological components and provides a global quality rating per study. The answer number per question is indicated between brackets.

| EPHPP Component question(s) Köhler  component et al. | Holden  et al. | Topper  et al. | Topper  et al. | Engsberg  et al. | Nayak  et al. | Torres-  Moreno | Houston  et al. | Tzeng  et al. | Ruder  et al. |
| --- | --- | --- | --- | --- | --- | --- | --- | --- | --- |
| (1989) ^3^^8^ | (1986) ^3^^9^ | (1990) ^40^ | (1990) ^42^ | (1992) ^4^^5^ | (2016) ^4^^8^ | et al. (1990) ^55^ | (1992) ^5^^0^ | (2015) ^44^ | (1992) ^4^^6^ |
| Selection bias  Selected individuals likely to be representative of Not likely | Not likely | Not likely | Not likely | Not likely | Can’t tell | Can’t tell | Not likely | Not likely | Not likely |
| target population? (3) | (3) | (3) | (3) | (3) | (4) | (5) | (3) | (3) | (3) |
| What percentage of selected individuals agreed to Can’t tell | Can’t tell | Can’t tell | Can’t tell | Can’t tell | Can’t tell | Can’t tell | Can’t tell | Can’t tell | Can’t tell |
| participate? (5) | (5) | (5) | (5) | (5) | (5) | (5) | (5) | (5) | (5) |
| Section rating Weak | Weak | Weak | Weak | Weak | Weak | Weak | Weak | Weak | Weak |
| Study design |  |  |  |  |  |  |  |  |  |
| Indicate the study design Cohort | Cohort | Cohort | Cohort | Cohort | Other: | Other: | Cohort | Cohort | Cohort |
| (5) | (5) | (5) | (5) | (5) | case (7) | case (7) | (5) | (5) | (5) |
| Study described as randomized? No | No | No | No | No | No | No | No | No | No |
| If yes, was the method of randomization NA | NA | NA | NA | NA | NA | NA | NA | NA | NA |
| described? |  |  |  |  |  |  |  |  |  |
| If yes, was the method appropriate? NA | NA | NA | NA | NA | NA | NA | NA | NA | NA |
| Section rating Moderate | Moderate | Moderate | Moderate | Moderate | Weak | Weak | Weak | Moderate | Moderate |
| Confounders |  |  |  |  |  |  |  |  |  |
| Were there important differences between groups Can’t tell | Can’t tell | Can’t tell | Can’t tell | Can’t tell | Can’t tell | Can’t tell | Can’t tell | Can’t tell | Can’t tell |
| prior to intervention? (3) | (3) | (3) | (3) | (3) | (3) | (3) | (3) | (3) | (3) |
| If yes, indicate the percentage of relevant NA | NA | NA | NA | NA | NA | NA | NA | NA | NA |
| confounders that were controlled (in design (e.g. |  |  |  |  |  |  |  |  |  |
| stratification, matching) or analysis) |  |  |  |  |  |  |  |  |  |
| Section rating Weak | Weak | Weak | Weak | Weak | Weak | Weak | Weak | Weak | Weak |
| Blinding |  |  |  |  |  |  |  |  |  |
| Was the outcome assessor aware of the Yes (1) | Yes (1) | Yes (1) | Yes (1) | Yes (1) | Can’t tell | Yes (1) | Yes (1) | Yes (1) | Yes (1) |
| intervention or exposure status of the participants? |  |  |  |  | (3) |  |  |  |  |
| Were the study participants aware of the research Yes (1) | Yes (1) | Yes (1) | Yes (1) | Yes (1) | Can’t tell | Yes (1) | Can’t tell | Yes (1) | Yes (1) |
| question? |  |  |  |  | (3) |  | (3) |  |  |
| Section rating Weak | Weak | Weak | Weak | Weak | Weak | Weak | Weak | Weak | Weak |
| Data collection methods |  |  |  |  |  |  |  |  |  |
| Were data collection tools shown to be valid? No (2) | No (2) | No (2) | No (2) | No (2) | Can’t tell | No (2) | No (2) | Yes (1) | No (2) |
|  |  |  |  |  | (3) |  |  |  |  |
| Were data collection tools shown to be reliable? No (2) | No (2) | No (2) | No (2) | No (2) | Can’t tell | No (2) | No (2) | Can’t tell | No (2) |
|  |  |  |  |  | (3) |  |  | (3) |  |
| Section rating Weak | Weak | Weak | Weak | Weak | Weak | Weak | Weak | Moderate | Weak |
| Withdrawals and drop-outs |  |  |  |  |  |  |  |  |  |
| Were withdrawals and drop-outs reported in terms Yes (1) | No (2) | Yes (1) | No (2) | No (2) | Can’t tell | No (2) | Yes (1) | No (2) | No (2) |
| of numbers and/or reasons per group? |  |  |  |  | (3) |  |  |  |  |
| Indicate the percentage of participants completing 80-100% | Can’t tell | 80-100% | Can’t tell | Can’t tell | Can’t tell | Can’t tell | Less than | Can’t tell | Can’t tell |
| the study (1) | (4) | (1) | (4) | (4) | (4) | (4) | 60% (3) | (4) | (4) |
| Section rating Strong | Weak | Strong | Weak | Weak | Weak | Weak | Weak | Weak | Weak |
| Global rating Weak | Weak | Weak | Weak | Weak | Weak | Weak | Weak | Weak | Weak |

**Table B1.** Details of quality assessment according to the Effective Public Health Practice Project (EPHPP) Quality Assessment Tool for Quantitative Studies (continued).

| EPHPP Component question(s) Aydin  component et al. | Steen  Jensen | Amrutsagar  et al. | Lemaire  et al. | Dean  et al. | Ö berg  et al. | Karakoç  et al. | Li et al.  (2019) ^5^^6^ | Ratto  et al. | Tang  et al. |
| --- | --- | --- | --- | --- | --- | --- | --- | --- | --- |
| (2018) ^54^ | et al. | (2020) ^53^ | (1999) ^51^ | (1985) ^4^^9^ | (1993) ^3^^7^ | (2017) ^4^^7^ |  | (2021) ^41^ | (2022) ^52^ |
|  | (2000) ^43^ |  |  |  |  |  |  |  |  |
| Selection bias  Selected individuals likely to be representative of Somewhat | Not likely | Not likely | Not likely | Not likely | Not likely | Somewhat | Not likely | Not likely | Can’t tell |
| target population? likely (2) | (3) | (3) | (3) | (3) | (3) | likely (2) | (3) | (3) | (5) |
| What percentage of selected individuals agreed to 80-100% | Can’t tell | Can’t tell | Can’t tell | Can’t tell | Can’t tell | Can’t tell | Can’t tell | Can’t tell | Can’t tell |
| participate? (1) | (5) | (5) | (5) | (5) | (5) | (5) | (5) | (5) | (5) |
| Section rating Moderate | Weak | Weak | Weak | Weak | Weak | Moderate | Weak | Weak | Weak |
| Study design  Indicate the study design Cohort | Cohort | Cohort | Cohort | Cohort | Cohort | Cohort | Cohort | Cohort | Other: |
| analytic | (5) | (5) | (5) | (5) | (5) | analytic | (5) | (5) | case (7) |
| Study described as randomized? No | No | No | No | No | No | (3)  No | No | No | No |
| If yes, was the method of randomization NA described?  If yes, was the method appropriate? NA | NA  NA | NA  NA | NA  NA | NA  NA | NA  NA | NA  NA | NA  NA | NA  NA | NA  NA |
| Section rating Moderate  Confounders  Were there important differences between groups No (2) | Moderate  Can’t tell | Moderate  Can’t tell | Weak  Can’t tell | Weak  Can’t tell | Moderate  Can’t tell | Moderate  No (2) | Moderate  Can’t tell | Moderate  Can’t tell | Weak  Can’t tell |
| prior to intervention? | (3) | (3) | (3) | (3) | (3) |  | (3) | (3) | (3) |
| If yes, indicate the percentage of relevant NA confounders that were controlled (in design (e.g. stratification, matching) or analysis)  Section rating Strong  Blinding  Was the outcome assessor aware of the Yes (1) intervention or exposure status of the participants?  Were the study participants aware of the research Yes (1) question?  Section rating Weak  Data collection methods  Were data collection tools shown to be valid? Yes (1) | NA  Weak Yes (1)  Yes (1) Weak  No (2) | NA  Weak Yes (1)  Yes (1) Weak  No (2) | NA  Weak Yes (1)  Yes (1) Weak  No (2) | NA  Weak Yes (1)  Yes (1) Weak  No (2) | NA  Weak Yes (1)  Yes (1) Weak  No (2) | NA  Strong Yes (1)  Yes (1) Weak  Yes (1) | NA  Weak Yes (1)  Yes (1) Weak  No (2) | NA  Weak Yes (1)  Yes (1) Weak  Yes (1) | NA  Weak Yes (1)  Yes (1) Weak  Yes (1) |
| Were data collection tools shown to be reliable? Yes (1) | No (2) | No (2) | No (2) | No (2) | No (2) | Yes (3) | No (2) | Yes (3) | Yes (3) |
| Section rating Strong  Withdrawals and drop-outs  Were withdrawals and drop-outs reported in terms NA of numbers and/or reasons per group?  Indicate the percentage of participants completing NA | Weak Yes (1)  80-100% | Weak No (2)  Can’t tell | Weak No (2)  Can’t tell | Weak No (2)  Can’t tell | Weak No (2)  Can’t tell | Strong No (2)  Can’t tell | Weak No (2)  Can’t tell | Strong No (2)  Can’t tell | Strong No (2)  Can’t tell |
| the study | (1) | (4) | (4) | (4) | (4) | (4) | (4) | (4) | (4) |
| Section rating Moderate | Strong | Weak | Weak | Weak | Weak | Weak | Weak | Weak | Weak |
| Global rating Moderate | Weak | Weak | Weak | Weak | Weak | Moderate | Weak | Weak | Weak |
